# Frailty Phenotype and Healthcare Costs in Older Community-Dwelling Men: The MrOS study

**DOI:** 10.1093/geroni/igaa057.2813

**Published:** 2020-12-16

**Authors:** Kristine Ensrud, Allyson Kats, Lisa Lisa Langsetmo, Tien Vo, John Schousboe

**Affiliations:** University of Minnesota, Minneapolis, Minnesota, United States

## Abstract

To determine the association of the frailty phenotype with subsequent healthcare costs, we studied 1514 men (mean age 79.3 years) participating in the 2007-2009 exam linked with their Medicare claims data. The frailty phenotype (5 components) was categorized as robust, pre-frail or frail. Multimorbidity and a frailty indicator (approximating the deficit accumulation index) were derived from claims data. Functional limitations were assessed by asking about difficulty performing 5 IADL. Total direct healthcare costs were ascertained during 36 months following the exam. Mean annualized costs (2018 dollars) was $5707 among robust, $8964 among pre-frail and $20,027 among frail men. Compared with robust, pre-frailty and frailty were each associated with higher costs after accounting for demographics, multimorbidity, functional limitations and the frailty indicator (cost ratio 1.18 [1.02-1.36] among pre-frail and 1.87 [1.47-2.39] among frail). Findings suggest that assessment of the phenotype may improve identification of individuals at increased risk of costly care.

